# Human Neutrophils Produce CCL23 in Response to Various TLR-Agonists and TNFα

**DOI:** 10.3389/fcimb.2017.00176

**Published:** 2017-05-12

**Authors:** Fabio Arruda-Silva, Francisco Bianchetto-Aguilera, Sara Gasperini, Sara Polletti, Emanuela Cosentino, Nicola Tamassia, Marco A. Cassatella

**Affiliations:** ^1^Section of General Pathology, Department of Medicine, University of VeronaVerona, Italy; ^2^Humanitas Clinical and Research CenterMilan, Italy; ^3^Department of Biomedical Sciences, Humanitas UniversityMilan, Italy; ^4^Functional Genomic Lab, Department of Biotechnology, University of VeronaVerona, Italy

**Keywords:** neutrophils, CCL23, R848, CCR1, IFN

## Abstract

CCL23, also known as myeloid progenitor inhibitory factor (MPIF)-1, macrophage inflammatory protein (MIP)-3, or CKβ8, is a member of the CC chemokine subfamily exerting its effects via CCR1 binding. By doing so, CCL23 selectively recruits resting T lymphocytes and monocytes, inhibits proliferation of myeloid progenitor cells and promotes angiogenesis. Previously, we and other groups have reported that human neutrophils are able to produce chemokines upon appropriate activation, including CCR1-binding CCL2, CCL3, and CCL4. Herein, we demonstrate that human neutrophils display the capacity to also express and release CCL23 when stimulated by R848 and, to a lesser extent, by other pro-inflammatory agonists, including LPS, Pam3CSK4, and TNFα. Notably, we show that, on a per cell basis, R848-activated neutrophils produce higher levels of CCL23 than autologous CD14^+^-monocytes activated under similar experimental conditions. By contrast, we found that, unlike CD14^+^-monocytes, neutrophils do not produce CCL23 in response to IL-4, thus indicating that they express CCL23 in a stimulus-specific fashion. Finally, we show that the production of CCL23 by R848-stimulated neutrophils is negatively modulated by IFNα, which instead enhances that of CCL2. Together, data extend our knowledge on the chemokines potentially produced by neutrophils. The ability of human neutrophils to produce CCL23 further supports the notion on the neutrophil capacity of orchestrating the recruitment of different cell types to the inflamed sites, in turn contributing to the control of the immune response.

## Introduction

Neutrophils are known to perform a series of effector functions, including phagocytosis, discharge of constitutively stored proinflammatory molecules, generation of massive amounts of superoxide anion, and active release of neutrophil extracellular traps (NETs), that are all crucial for innate immunity responses toward infections (Scapini et al., 2013). In addition to their defensive functions, neutrophils display the capacity to synthesize and secrete a variety of cytokines, including many chemokines (Tecchio and Cassatella, 2016). For instance, depending on the stimulatory conditions, human neutrophils have been shown to produce both CXC chemokines, such as CXCL1 (GROα), CXCL3, CXCL5 (ENA78), CXCL6, CXCL8 (IL-8), CXCL9 (MIG), CXCL10 (IP-10), CXCL11 (I-TAC), and CC chemokines, including CCL2 (MCP-1), CCL3 (MIP-1α), CCL4 (MIP-1β), CCL17, CCL18, CCL19 (MIP-3β), and CCL20 (MIP-3α) (reviewed by Tecchio and Cassatella, 2016). Given that these chemokines recruit leukocytes associated to either the innate [including neutrophils themselves, monocytes, macrophages, dendritic cells (DCs), and natural killer (NK) cells] or the acquired (such as T cell subsets) immune responses (Viola and Luster, 2008; Griffith et al., 2014), it is plausible to speculate that neutrophils are in the position to orchestrate leukocyte trafficking during the entire course of specific responses to invading pathogens or other antigens (Tecchio and Cassatella, 2016).

In this context, CCL23, also known as myeloid progenitor inhibitory factor (MPIF)-1, macrophage inflammatory protein (MIP)-3, or CKβ8, is a recently identified CCL, which, similarly to CCL3, CCL5, CCL7, CCL13, CCL14, CCL15, and CCL16, binds CCR1 (Youn et al., 1998; Viola and Luster, 2008). Also CCL4 and CCL2 have been shown to bind CCR1: the former one, however, after natural truncation of its two NH2-terminal amino acids (Guan et al., 2002), the latter one with lower affinity as compared to its binding to CCR2 (Menten et al., 2002). Similarly to other CCR1 binding chemokines, CCL23 exerts chemotactic activities on monocytes, DCs and resting T lymphocytes (Patel et al., 1997; Youn et al., 1998; Nardelli et al., 1999), as well as on endothelial cells to ultimately induce tube formation (Hwang et al., 2005; Son et al., 2006). Moreover, CCL23 has been reported to play a role in bone formation, for its potent chemoattractant action on osteoclast precursors, but not for fully differentiated osteoclasts or osteoblasts (Votta et al., 2000). A more precise characterization of CCL23 function is however hindered by the fact that CCL23 is a gene with no mouse ortholog (Viola and Luster, 2008), which thus prevents the employment of loss-of-function strategies in experimental animal models. While CCL23/MPIF-1 cDNA was originally isolated from a human aortic endothelial library (Patel et al., 1997), subsequent analysis made on various cell lines has shown that CCL23 mRNA is readily detectable in myelomonocytic cell lines (Patel et al., 1997). Expression of CCL23 mRNA was then found in monocytes stimulated with IL-1β (Forssmann et al., 1997) and it is now established that IL-4-treated monocytes produce remarkable amounts of CCL23 (Nardelli et al., 1999; Novak et al., 2007). CCL23 can be detected in synovial fluids from rheumatoid arthritis patients (Berahovich et al., 2005), as well as in serum of systemic sclerosis patients (Yanaba et al., 2011). More recently, eosinophils freshly isolated from blood were found to express CCL23 mRNA and, in turn, secrete antigenic CCL23 upon incubation with IL-5 or GM-CSF (Matsumoto et al., 2011).

Since no information was present in the literature on an eventual expression/production of CCL23 by neutrophils, we decided to investigate such a possibility. As a result, we herein show that also human neutrophils produce CCL23 *in vitro*, yet upon incubation with selected stimuli and in a fashion regulated by endogenous TNFα and exogenous IFNα.

## Materials and methods

### Cell purification and culture

Granulocytes and autologous CD14^+^-monocytes were isolated from buffy coats of healthy donors, under endotoxin-free conditions as previously described (Davey et al., 2011). Briefly, buffy coats were stratified on Ficoll-Paque PLUS gradient (GE Healthcare, Little Chalfont, United Kingdom) at a 1:1 ratio, and then centrifuged at 400 × g for 30 min at room T. Granulocytes were then collected and subjected to dextran sedimentation followed by hypotonic lysis to remove erythrocytes. Finally, neutrophils were isolated from granulocytes (to reach a 99.7 ± 0.2% purity) by positively removing all contaminating cells using the EasySep neutrophil enrichment kit (StemCell Technologies, Vancouver, Canada; Calzetti et al., 2017). CD14^+^-monocytes were instead isolated from PBMCs, obtained after Ficoll-Paque gradient centrifugation, by anti-CD14 microbeads (Miltenyi Biotec, Bergisch Gladbach, Germany) to reach a purity of ~98%. Cells were then suspended at 5 × 10^6^/ml in RPMI 1640 medium supplemented with 10% low endotoxin FBS (<0.5 EU/ml; from BioWhittaker-Lonza, Basel, Switzerland) and then plated either in 6/24-well tissue culture plates or in polystyrene flasks (from Greiner Bio-One, Kremsmünster, Austria) for culture at 37°C, 5% CO_2_ atmosphere, in the presence or the absence of: 0.2–50 μM R848, 5–50 μM R837, 1 μg/ml Pam3CSK4 (Invivogen, San Diego, CA, USA), 10 ng/ml TNFα (Peprotech, Rocky Hill, NJ, USA), 0.1–10 μg/ml ultrapure LPS from *E. coli* 0111:B4 strain (Alexis, Enzo Life Sciences, Farmingdale, NY, USA), 1,000 U/ml pegylated IFNα-2a (Pegasys, Roche, Basel, Switzerland), 100 U/ml IFNγ (R&D Systems, Minneapolis, MN, USA), 10 ng/ml GM-CSF (Miltenyi Biotec), 1,000 U/ml G-CSF (Myelostim, Italfarmaco Spa, Milano, Italy), 10–100 ng/ml IL-18 (MBL International, Nagoya, Japan), 10–100 ng/ml IL-33 (Peprotech), 10 nM fMLF (Sigma, Saint Louis, MO, USA), 500 μg/ml particulate β-glucan (InvivoGen), or 500 μg/ml curdlan (Sigma). In some experiments, neutrophils were preincubated for 30 min with either 10 μg/ml adalimumab (Humira, Abbott Biotechnology Limited, Barceloneta, Puerto Rico) or 10 μg/ml etanercept (Enbrel, Amgen, Thousand Oaks, CA, USA), prior to further incubation with stimuli. After the desired incubation period, neutrophils and monocytes were collected and spun at 300 × g for 5 min. Cell-free supernatants were immediately frozen in liquid nitrogen and stored at −80 °C, while the corresponding cell pellets were either extracted for total RNA or lysed for protein analysis.

### ELISA

Cytokine concentrations in cell-free supernatants and cell-lysates were measured by commercially available ELISA kits, specific for human: CXCL8 (Mabtech, Nacka Strand, Sweden), CCL23 (R&D Systems, Minneapolis, MN, USA), CCL2 (R&D Systems), CCL3 (eBioscience, San Diego, CA, USA), and CCL4 (eBioscience). Detection limits of these ELISA were: 7.8 pg/ml for CCL23, 7.8 pg/ml for CCL2, 16 pg/ml for CCL3, 4 pg/ml for CCL4, and 16 pg/ml for CXCL8.

### Reverse transcription quantitative real-time PCR (RT-qPCR)

Total RNA was extracted from neutrophils and monocytes by the RNeasy Mini Kit (Qiagen, Venlo, Limburg, Netherlands), according to the manufacturer's instructions. An on-column DNase digestion with the RNase-free DNase set (Qiagen) was also performed during total RNA isolation to completely remove any possible contaminating DNA. Purified total RNA was then reverse-transcribed into cDNA using Superscript III (Life Technologies) and random hexamer primers (Life Technologies), while qPCR was carried out using Fast SYBR® Green Master Mix (Life Technologies) (Tamassia et al., 2014). Sequences of the gene-specific primers (Life Technologies) used in this study are the following: GAPDH, forward AACAGCCTCAAGATCATCAGC and reverse GGATGATGTTCTGGAGAGCC; CXCL8 forward CTGGCCGTGGCTCTCTTG and reverse CCTTGGCAAAACTGCACCTT; IL-1ra forward TTCCTGTTCCATTCAGAGACGAT and reverse AATTGACATTTGGTCCTTGCAA; CCL2 forward GTCTCTGCCGCCCTTCTGT and reverse TTGCATCTGGCTGAGCGAG; CCL3 forward AGCCCACATTCCGTCACCTG and reverse CGTGTCAGCAGCAAGTGATG; CCL4 forward CGCCTGCTGCTTTTCTTACAC and reverse CAGACTTGCTTGCTTCTTTTGG; and CCL23 forward GTACTTCTGGACATGCTCTGG and reverse CTGAACTTGCTTATCACTGGG. Data were calculated by Q-Gene software (http://www.gene-quantification.de/download.html) and expressed as mean normalized expression (MNE) units after GAPDH normalization.

### RNA sequencing (RNA-seq)

Total RNA extracted from neutrophils (50 × 10^6^/condition) was quality checked by Agilent 2100 Bioanalyzer (Agilent Technologies). RNA integrity (RIN) was routinely found to be ≥7.0. RNA-seq libraries were prepared by oligo-dT selection using the TruSeq RNA sample preparation kit (Illumina, San Diego, CA, USA) and sequenced on an Illumina HiSeq 2000. After quality filtering according to the Illumina pipeline, 51-base-pair (bp) reads were aligned to the hg19 assembly (Genome Reference Consortium GRCh37) as well as the human transcriptome reference (UCSC annotation), using TopHat (Trapnell et al., 2013). We allowed up to two mismatches and specified a mean distance between pairs (–r) of 250 bp. The reference sequence and annotation files were downloaded from iGenomes repository at the following website: http://support.illumina.com/sequencing/sequencing_software/igenome.html. Read counts per gene were obtained from the aligned reads using “htseq-count” command from the HTSeq framework version 0.6.1p1 (Anders et al., 2015), with the following parameters: “–f bam –r name –m union –s no –t exon –i gene id”. Count normalization was performed using the Bioconductor/R packages DESeq2 (Love et al., 2014). Transcript abundance was indicated as Fragments Per Kilobase of transcript sequence per Million of mapped fragments (FPKM), using the “fpkm” function of DESeq2.

### Statistical analysis

Data are expressed as mean ± SEM. Statistical evaluation was performed by the Student's *t*-test, one-way ANOVA followed by Tukey's *post-hoc* test or two-way ANOVA followed by Bonferroni's *post-hoc* test. Values of *p* < 0.05 were considered as statistically significant.

### Study approval

This study was carried out in accordance with the recommendations of “Ethic Committee of the Azienda Ospedaliera Universitaria Integrata di Verona (Italy)” with written informed consent from all subjects. All subjects gave written informed consent in accordance with the Declaration of Helsinki.

## Results

### Human neutrophils incubated with R848 accumulate CCL23 transcripts

**Figure 1:**
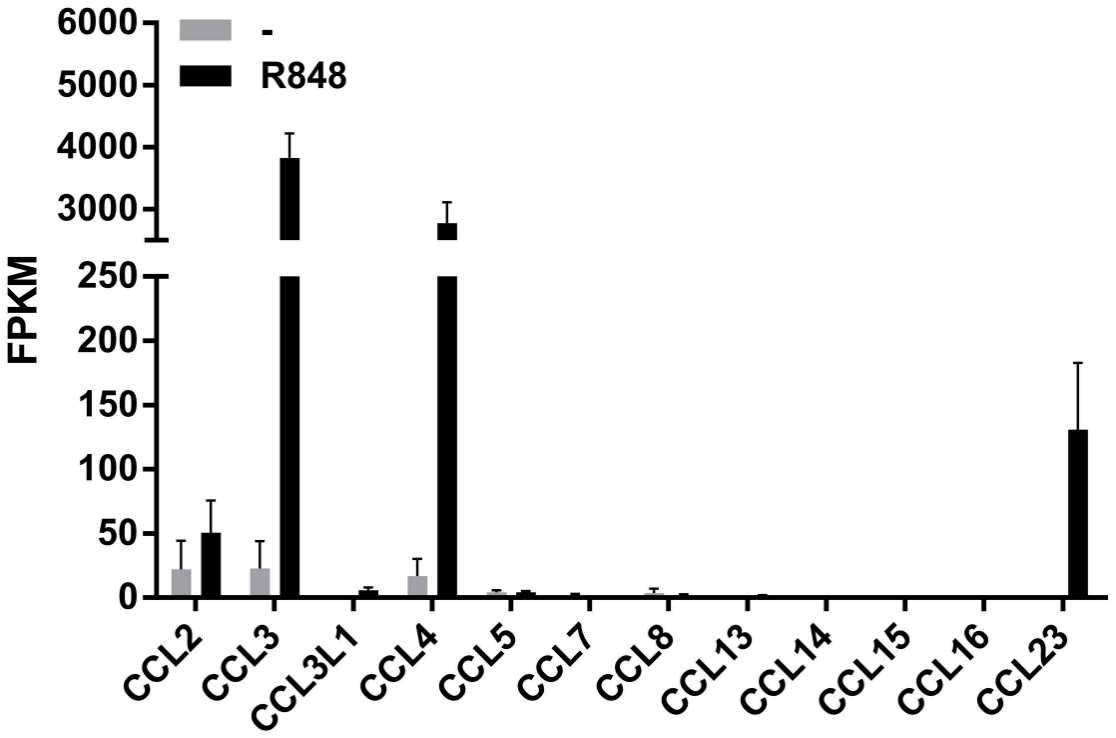
**Expression of CCR1-binding chemokine mRNAs in human neutrophils incubated with R848**. Human neutrophils were incubated with or without 5 μM R848 for 24 h, and then total RNA was extracted and processed for RNA-seq experiments. Data are presented as FPKM (fragment per kilobase of exons per million fragments mapped) from two independent experiments (mean ± SEM).

We have recently reported that R848 represents a very powerful inducer of IL-6, TNFα, G-CSF, IL-12-p40, and CXCL8 production by human neutrophils (Zimmermann et al., 2015, 2016). We recall here that R848 is an imidazoquinoline compound that potently activates the signaling of both TLR7 and TLR8 (Jurk et al., 2002), but that in human neutrophils activates only TLR8 (Janke et al., 2009; Zimmermann et al., 2015) as these cells do not express TLR7 (Hayashi et al., 2003; Janke et al., 2009; Berger et al., 2012; Zimmermann et al., 2015). Moreover, results from RNA-seq experiments to discover unidentified molecules eventually generated by human neutrophils incubated for 24 h with 5 μM R848 indicated that, among the various CCR1-binding chemokines (Griffith et al., 2014), neutrophils express the mRNA for CCL2, CCL3, CCL4, and CCL23, but not CCL5, CCL7, CCL8, CCL13, CCL14, CCL15, and CCL16 (Figure 1). Figure 1 further shows that, in R848-stimulated neutrophils, the levels of CCL3 and CCL4 mRNAs are remarkably higher than those of CCL2 and CCL23 mRNAs. While neutrophil expression of CCL2, CCL3, and CCL4 mRNA is well-established (Kasama et al., 1993, 1994; Cassatella, 1999; Yamashiro et al., 1999), that of CCL23 has been never described before.

**Figure 2:**
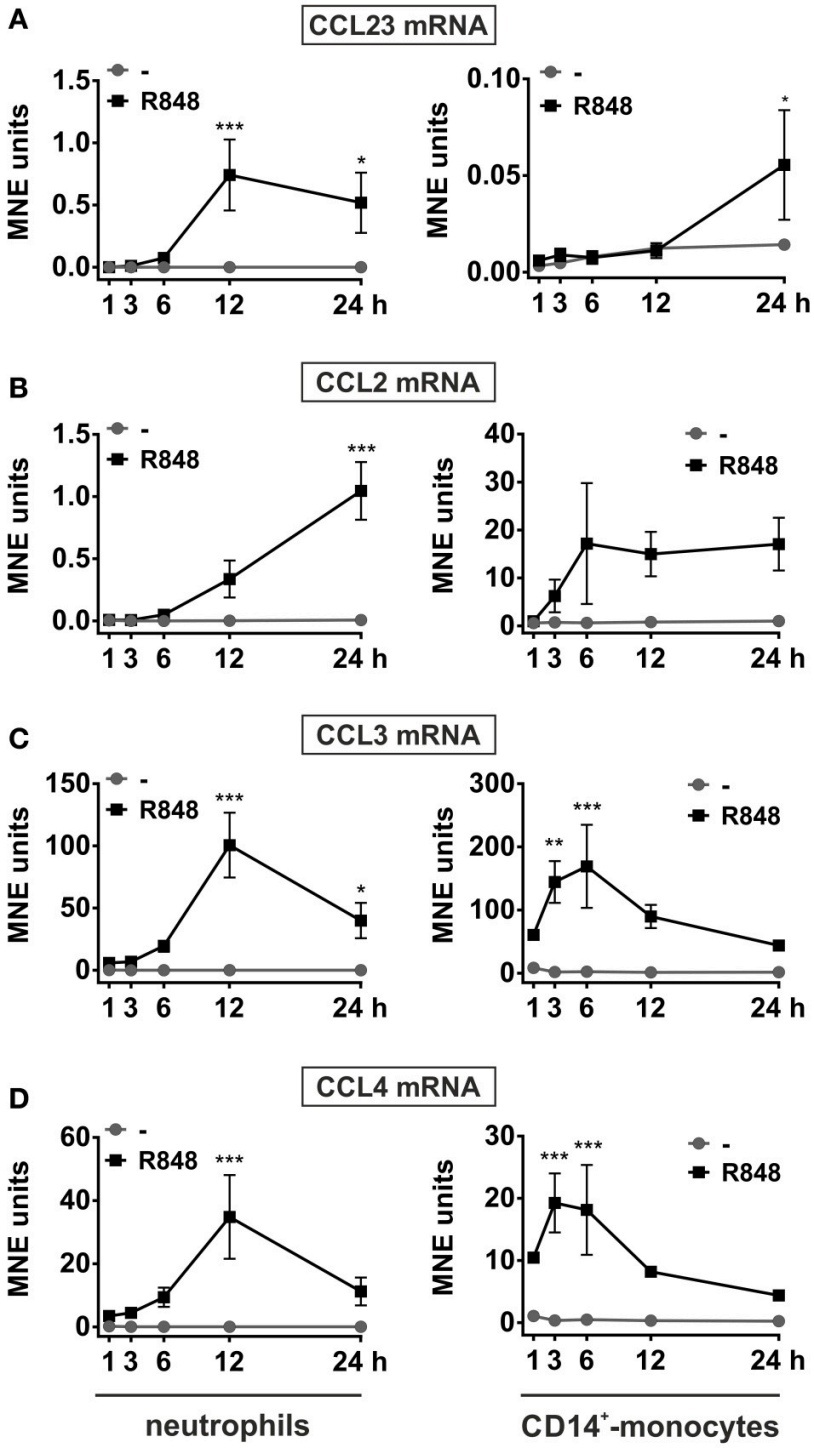
**Kinetics of CCL23, CCL2, CCL3, and CCL4 mRNA expression in neutrophils and autologousCD14^**+**^-monocytes incubated with R848**. Neutrophils and autologous CD14^+^-monocytes were cultured with or without 5 μM R848 for up to 24 h to evaluate their CCL23 **(A)**, CCL2 **(B)**, CCL3 **(C)**, and CCL4 **(D)** mRNA expression by RT-qPCR. Gene expression is depicted as mean normalized expression (MNE) units after GAPDH mRNA normalization (mean ± SEM, *n* = 5). Asterisks stand for significant increase: ^*^P < 0.05, ^**^P < 0.01, ^***^P < 0.001, by two-way ANOVA followed by Bonferroni's post-test.

To validate and extend these preliminary findings, neutrophils, as well as autologous CD14^+^-monocytes, incubated with R848 were subjected to RT-qPCR experiments in which the kinetics of CCL23, CCL2, CCL3, and CCL4 mRNA expression were compared. As shown in Figure 2, CCL23 mRNA expression in neutrophils started to be detected after 3 h, peaked at the 12 h-time point and slowly declined thereafter (Figure 2A, left panel). Similarly to CCL23, also CCL2 mRNA started to be accumulated in neutrophils later than 3 h, but then steadily increased up to 24 h (Figure 2B, left panel). By contrast, CCL3 and CCL4 mRNAs were induced by R848 as early as after 1 h, but then followed a time-course pattern similar to CCL23 mRNA (Figures 2C,D, left panels). Interestingly, maximum expression levels of both CCL23 and CCL2 mRNAs in R848-treated neutrophils were similar (Figures 2A,B, left panels), but much lower than those of CCL3 and CCL4 mRNAs (Figures 2C,D, left panels). Notably, expression levels, as well as kinetics of CCL23 (Figure 2A, right panel), CCL2 (Figure 2B, right panel), CCL3 (Figure 2C, right panel), and CCL4 (Figure 2D, right panel) mRNAs in R848-treated monocytes strikingly differed from those in neutrophils. For instance, the peak of CCL2, CCL3, and CCL4 mRNA expression in R848-treated monocytes was found to occur earlier than in neutrophils (Figures 2B,C), unlike that of CCL23 mRNA, which was found to take place later, consistent with the increase of CCL23 transcripts starting not before 12 h of incubation (Figure 2A). Moreover, while CCL2 and CCL3 mRNAs accumulated in R848-treated monocytes at higher levels than in neutrophils (Figures 2B,C), the opposite was observed in the case of CCL23 and CCL4 transcripts (Figures 2A,D). Altogether, data demonstrate that neutrophils incubated with R848 genuinely express CCL23 mRNA, yet in a different fashion as compared to CCL2, CCL3, and CCL4 genes, or to CCL23 mRNA expression induced in autologous monocytes.

### Human neutrophils incubated with R848 produce discrete levels of CCL23

**Figure 3:**
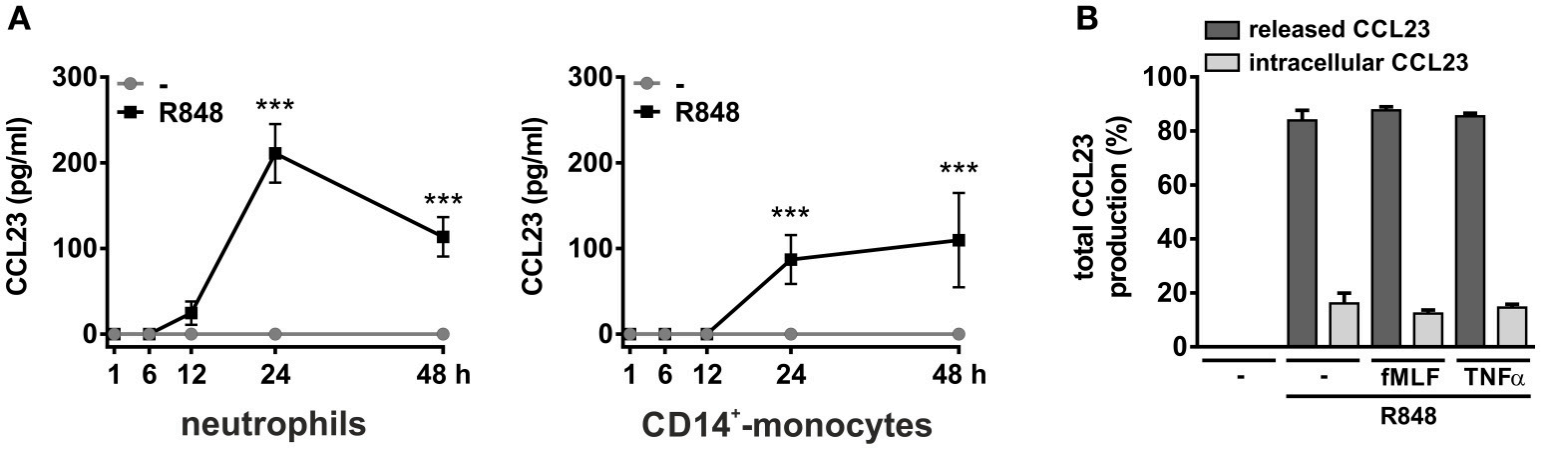
**Production of CCL23 by neutrophils and monocytes incubated with R848**. Neutrophils and CD14^+^-monocytes were cultured at 5 × 10^6^ cells/ml with or without 5 μM R848 for up to 48 h to evaluate their capacity to produce and release CCL23, as detected by ELISA (*n* = 4–19) **(A)**. In **(B)**, neutrophils were cultured with or without 5 μM R848 for 24 h and then further incubated for 4 h with or without 10 nM fMLF or 10 ng/ml TNFα to evaluate released and cell-associated CCL23 (*n* = 4). Asterisks stand for significant increases: ^***^P < 0.001 by two-way ANOVA followed by Bonferroni's post-test.

Next, we analyzed whether R848-treated neutrophils are able to synthesize and release CCL23 and found that they actually do so. CCL23 protein was, in fact, detected in neutrophil-derived supernatants, but starting only after 12 h of cell incubation with R848, and with peak levels reached after 24 h (211.1 ± 34.2, *n* = 4–13) (Figure 3A, left panel). This is consistent with the delayed induction of CCL23 mRNA by R848, which, by the way, is also known to prolong neutrophil survival (Zimmermann et al., 2016). Moreover, previous dose-response experiments revealed that 5 μM R848 correspond to the most effective concentrations to induce the production of CCL23 (data not shown). We also observed that, of the total CCL23 synthesized by R848-treated neutrophils, ~15% remains cell-associated (Figure 3B), indicating that CCL23 is almost completely released. And in fact, two potent secretagogues, namely fMLF or TNFα, were found unable to mobilize the small quote of intracellular CCL23 accumulated in R848-treated neutrophils (Figure 3B).

**Figure 4:**
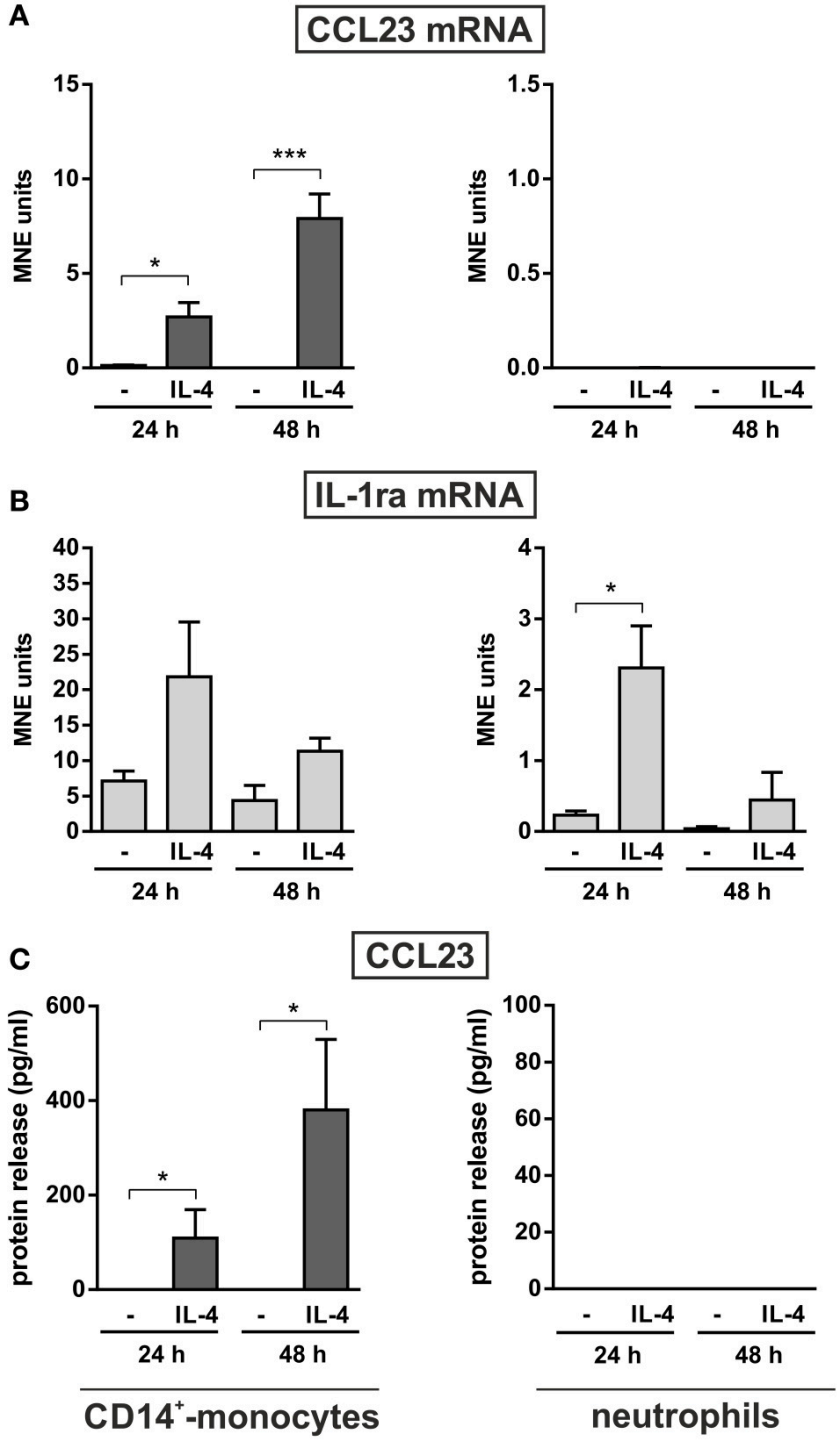
**Effect of IL-4 on the production of CCL23 by monocytes and neutrophils**. CD14^+^-monocytes and neutrophils were cultured at 5 × 10^6^ cells/ml with or without 20 ng/ml IL-4 for 24 and 48 h to evaluate their CCL23 **(A)** and IL-1ra **(B)** mRNA expression by RT-qPCR, as well as their CCL23 release **(C)** by ELISA (*n* = 4). Gene expression data are depicted as MNE units after GAPDH mRNA normalization (mean ± SEM). Asterisks stand for significant increases: ^*^P < 0.05, ^***^P < 0.001 by two-way ANOVA followed by Bonferroni's post-test.

Monocytes too were found to release CCL23 in response to R848 (Figure 3A, right panel), surprisingly at lower levels (87.1 ± 28.5, *n* = 11) than neutrophils, but consistent with the gene expression data (Figure 2A). However, monocytes incubated with IL-4 for up to 48 h were found to accumulate and release remarkable levels of CCL23 mRNA and protein (308.6 ± 149.2 pg/ml, *n* = 4) (left panels in Figure 4A and Figure 4C, respectively), confirming previous data (Novak et al., 2007). By contrast, IL-4-treated neutrophils did not express/produce any CCL23 mRNA and protein (right panels in Figure 4A and Figure 4C, respectively), even though they accumulated IL-1ra mRNA (right panel in Figure 4B), as previously described (Crepaldi et al., 2002). Altogether, data prove that neutrophils genuinely produce CCL23 in response to R848 but not to IL-4.

### Effect of IFNα on CCL23 production by R848-treated neutrophils

**Figure 5:**
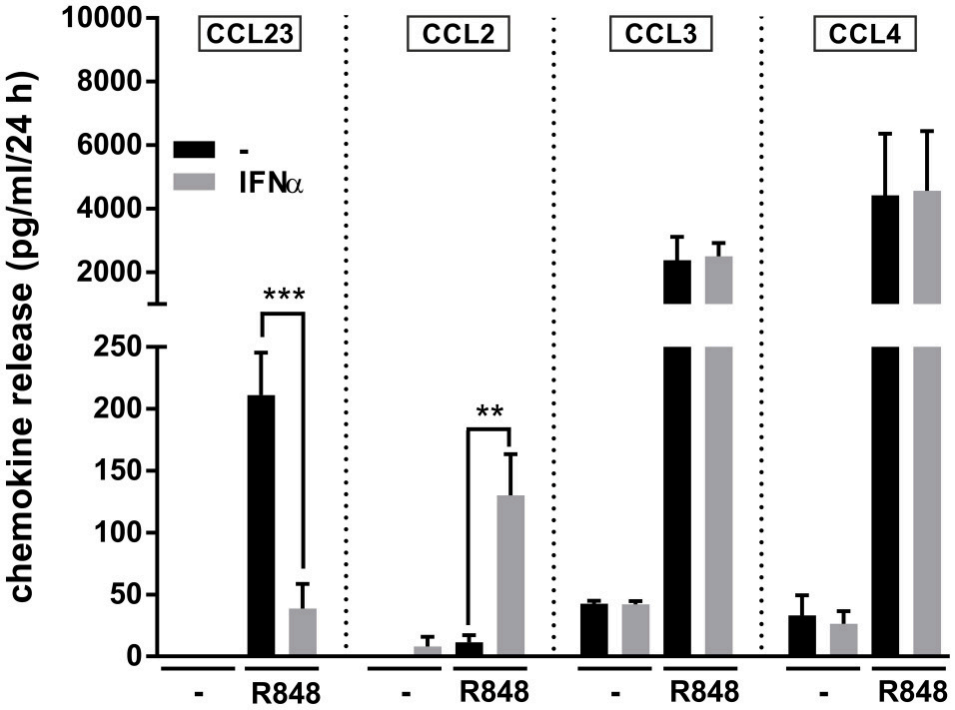
**IFNα downregulates the production of CCL23 by R848-treated neutrophils** Neutrophils (5 × 10^6^/ml) were cultured with or without 1,000 U/ml IFNα and/or 5 μM R848. After 24 h, supernatants were collected and subjected to CCL23, CCL2, CCL3, and CCL4 measurement by ELISA. Data are depicted as mean ± SEM (*n* = 3–19). Asterisks stand for significant increases: ^**^P < 0.01, ^***^P < 0.001, by two-way ANOVA followed by Bonferroni's post-test.

Given the recently reported capacity of IFNα to potently increase IL-6 and TNFα mRNA and protein expression in R848-treated neutrophils (Zimmermann et al., 2016), we investigated IFNα-mediated effects on neutrophil-derived CCL23 and other CCR1-binding chemokines. As displayed in Figure 5, we found that, while IFNα strongly downregulates the production of CCL23 by neutrophils incubated with R848 for 24 h, it significantly upregulates that of CCL2, but does not influence CCL3 and CCL4 release. Interestingly, Figure 5 also illustrates that the amounts of CCL3 and CCL4 released by R848-stimulated neutrophils are higher than those of CCL2 and CCL23, in accordance with the gene expression data (Figure 2). Taken together, data provide evidence that IFNα negatively modulates CCL23, but not CCL2, CCL3, and CCL4 production by R848-treated neutrophils.

### Other TLR agonists, as well as TNFα, trigger the production of CCL23 by human neutrophils

**Figure 6:**
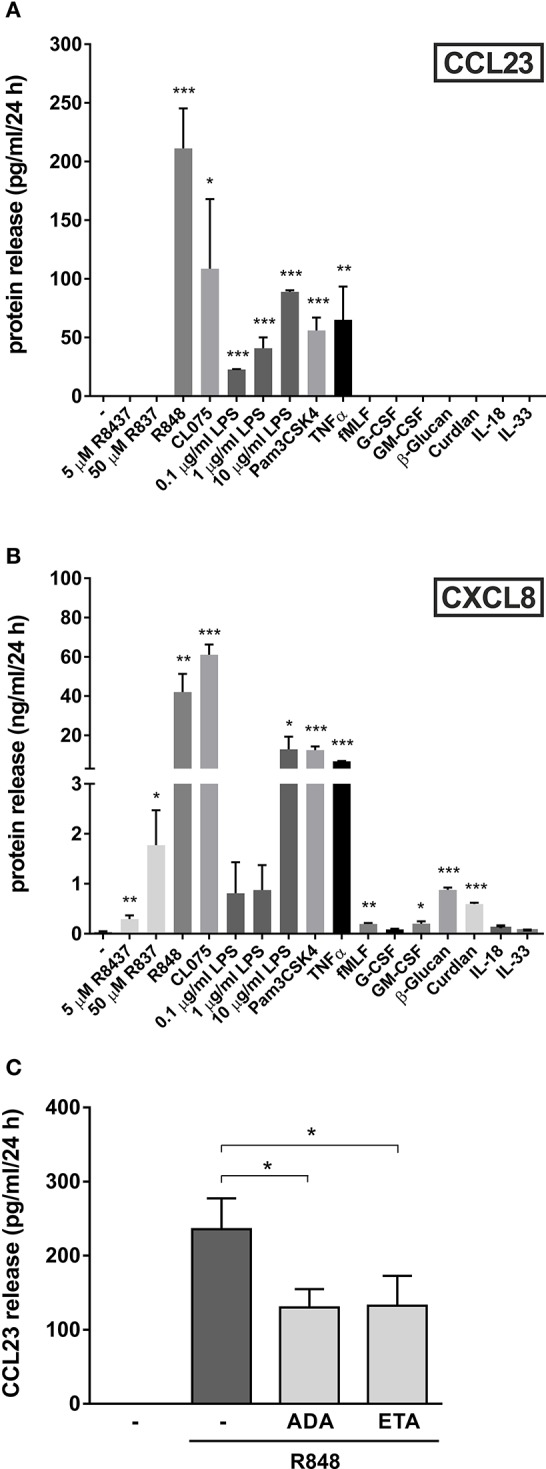
**Other agonists, besides R848, trigger the production of CCL23 by human neutrophils**. Neutrophils (5 × 10^6^/ml) were cultured with or without 5–50 μM R837, 5 μM R848, 5 μM CL075, 0.1–10 μg/ml LPS, 1 μg/ml Pam3CSK4, 10 ng/ml TNFα, 10 nM fMLF, 10 ng/ml G-CSF, 10 ng/ml GM-CSF, 500 μg/ml β-Glucan, 500 μg/ml Curdlan, 100 ng/ml IL-18, and 100 ng/ml IL-33 for 24 h. Then, extracellular supernatants were collected to evaluate CCL23 **(A)** and CXCL8 **(B)** production by ELISA (mean ± SEM, *n* = 3–5). Asterisks stand for significant increases as compared to untreated cells: ^*^P < 0.05, ^**^P < 0.01, ^***^P < 0.001, by Student's *t*-test. **(C)** Neutrophils (5 × 10^6^/ml) were pretreated for 30 min with 10 μg/ml adalimumab (ADA) or 10 μg/ml etanercept (ETA), to be subsequently incubated for further 24 h with 5 μM R848. Cell-free supernatants were then collected to evaluate CCL23 production by ELISA (mean ± SEM, *n* = 3–5). Asterisks stand for significant inhibition: ^*^P < 0.05, by one-way ANOVA followed by Tukey's post-test.

In a final series of experiments, we investigated whether other agonists could induce the production of CCL23 by human neutrophils. We found that, in addition to R848 (the most potent one), also 5 μM CL075 (another ligand for TLR8), 1 μg/ml Pam3CSK4 (a ligand for TLR2), 10 μg/ml ultrapure LPS (a ligand for TLR4), and 10 ng/ml TNFα promoted the production of remarkable amounts of CCL23 by neutrophils (respectively, 108.6 ± 59.3, 61.8 ± 13.2, 88.8 ± 1.3, and 65.1 ± 28.2 pg/ml, *n* = 3–5) (Figure 6A). By contrast, 5–50 μM R837 (a ligand for TLR7), 500 μg/ml β-glucan and 500 μg/ml curdlan (both ligands for Dectin-1), 10 nM fMLF, 10 ng/ml GM-CSF, and 1,000 U/ml G-CSF, all failed to trigger any extracellular CCL23 production by neutrophils (Figure 6A), albeit able to stimulate the production of CXCL8 (Figure 6B). Finally, 100 ng/ml IL-18 and IL-33, two cytokines having potential roles in Th2-responses, resulted unable to induce CCL23 production in both human neutrophils (Figure 6A) and CD14^+^-monocytes (data not shown). IL-18 and IL-33 also did not modulate the levels of CCL23 produced by R848-treated neutrophils (data not shown).

Given that R848-treated neutrophils are known to produce elevated amounts of TNFα (Zimmermann et al., 2015), we then investigated whether CCL23 production could be amplified by endogenous TNFα, as occurring in the case of IL-6 (Zimmermann et al., 2015). This was found to be the case, as adalimumab or etanercept, two potent TNFα-neutralizing drugs (Tracey et al., 2008), diminished the detection of extracellular CCL23 in supernatants from neutrophils treated with R848 for 24 h by, respectively, 44.7 ± 5.5% (*n* = 3) and 45.7 ± 11.4% (*n* = 3) (Figure 6C). Altogether, data uncover that neutrophils produce CCL23 in response to discrete stimuli. Data also demonstrate that, at least in the case of R848-treated neutrophils, CCL23 production is amplified *via* endogenous TNFα.

## Discussion

Neutrophils are among the first cell types that infiltrate inflammatory sites. As such, they may play an important role in coordinating the subsequent recruitment of other leukocytes *via* the generation of a variety of chemoattractants, including chemokines (Tecchio and Cassatella, 2016). Among the latter molecules, neutrophils have been already shown to represent sources of some CCR1-binding chemokines, namely CCL3 and CCL4 (Kasama et al., 1994; Scapini et al., 2000), known to act on monocytes, DCs and resting T lymphocytes (Patel et al., 1997; Nardelli et al., 1999; Viola and Luster, 2008).

In this study, we show that human neutrophils are able to produce another CCR1-binding chemokine, namely CCL23, in response to ligands for TLR2 (Pam3CSK4), TLR4 (LPS), and much more efficiently, TLR8 (R848 and CL075). By contrast, a variety of inflammatory mediators previously identified as being effective stimuli for the production of chemokines by neutrophils, for instance G-CSF, GM-CSF, and fMLF (Cassatella, 1999), failed to trigger CCL23 production. Other CCR1-binding chemokines, including CCL5, CCL7, CCL13, CCL14, CCL15, and CCL16 were found as not induced in neutrophils, at least after R848-treatment. Surprisingly, the amounts of CCL23 produced by neutrophils incubated with R848 for 24 h were comparable to those made by the same number of autologous R848-treated monocytes. This finding is quite unusual because, on a per cell basis, activated monocytes usually produce higher cytokine/chemokine levels than autologous neutrophils (Cassatella, 1999; Dale et al., 2008), although some other exceptions exist, for instance CCL19/MIP3β (Scapini et al., 2001). Whatever the case is, our findings exclude that the observed CCL23 production by R848-treated neutrophils derives from potential contaminating monocytes. Moreover, they also imply that appropriately activated neutrophils may represent major sources of CCL23, even in consideration of the fact that, during bacterial or viral infections, neutrophils often outnumber mononuclear leukocytes by one to two orders of magnitude.

We also report that, differently from autologous monocytes (Novak et al., 2007), neutrophils do not produce CCL23 upon incubation with IL-4, a Th2 cytokine, even though they promptly accumulate IL-1ra mRNA (Re et al., 1993; Crepaldi et al., 2002). Although we do not have a formal molecular explanation for the inability of IL-4-treated neutrophils to express CCL23 mRNA yet, our preliminary genome-wide map of both histone H3 monomethylated at K4 (H3K4me1, which is associated with active or poised genomic regulatory elements) (Smale et al., 2014) and PU.1 (an Ets-family transcription factor marking the majority of regulatory elements in myeloid cells) (Smale et al., 2014) would reveal that neutrophils contain only one regulatory region at the *CCL23* locus (1 kb upstream from *CCL23* TSS), while monocytes contain two ones (1 and 25 kb upstream from *CCL23* TSS). Such a different chromatin conformation might be responsible for the differential ability of neutrophils and monocytes to express CCL23 mRNA in response to IL-4 stimulation, as the “closed” chromatin conformation in neutrophils would, in fact, prevent the binding of IL-4-activated transcription factor(s) to it, and consequently the transcription of CCL23 mRNA. Interestingly, other Th2 cytokines, namely IL-18 and IL-33, were found unable to trigger the production of CCL23 by human neutrophils. Therefore, along with the findings on IL-4, our data would exclude the generation of neutrophils conditioned by Th2 cytokines and associated to Th2-microenvironment.

According to previous findings (Youn et al., 1998), CCL23 may exist in multiple forms arising from both alternative splicing and post-translational processing. CCL23 cDNA encodes, in fact, a signal sequence of 21 aa followed by either a 99 aa (also known as CKβ8) or 116 aa (CKβ8-1) mature proteins, which both represent putative ligands for CCR1 (Youn et al., 1998). In such regard, our preliminary RT-qPCR experiments would indicate that R848-stimulated neutrophils express both mRNA isoforms, while monocytes express only CKβ8-1 mRNA (NT, FA, and MAC, unpublished results). As mentioned, eosinophils have been shown to express and release CCL23, and, similarly to R848-activated neutrophils, they accumulate both mRNA isoforms of CCL23 (Matsumoto et al., 2011). In any case, we exclude that the two CCL23 mRNAs detected in neutrophils derive from potential contaminating eosinophils, as our neutrophil purification method reduces eosinophil contamination below 0.1% (Calzetti et al., 2017). Moreover, GM-CSF, which triggers the release of CCL23 by eosinophils (Matsumoto et al., 2011), resulted ineffective in neutrophils.

In this work, we also report that the production of CCR1-binding chemokines by R848-activated neutrophils is differentially modulated by IFNα. In fact, while production of CCL3 and CCL4 was not affected by the addition of IFNα to neutrophils, that of CCL23 was strongly inhibited. By contrast, CCL2 production was strongly enhanced in neutrophils incubated with R848 in the presence of IFNα, similarly to what previously observed in neutrophils treated with IFNγ plus LPS (Yoshimura and Takahashi, 2007). Together, our data therefore reinforce the notion that type I interferons do not act as mere enhancers of inflammatory gene expression, but represent instead fine tuners regulating the transcription of a variety of mediators (González-Navajas et al., 2012; McNab et al., 2015), including neutrophil-derived chemokines. Given the potent effect of TLR8 agonists in inducing neutrophil-derived CCL23, and its negative regulation by IFNα, data also contribute to extend our knowledge on the complex role of neutrophils to both host defense and disease in response to viral infections (Tamassia and Cassatella, 2013; Galani and Andreakos, 2015).

## Author contributions

All authors were involved in discussing and drafting the article, approved the final version to be published, and had full access to all data, taking responsibility for their integrity and analysis accuracy. FA, SG, SP, EC, and NT performed the experiments, FA, FB, NT, and MC analyzed the results, FA, NT, and MC conceived the experiments and wrote the paper.

## Funding

This work was supported by grants from Associazione Italiana per la Ricerca sul Cancro (AIRC, IG-15454) to MC and Ministero dell'Istruzione, dell'Università e della Ricerca (PRIN 2015YYKPNN). FA is supported by Brazilian fellowship from Coordenação de Aperfeiçoamento de Pessoal de Nível Superior (CAPES).

### Conflict of interest statement

The authors declare that the research was conducted in the absence of any commercial or financial relationships that could be construed as a potential conflict of interest.

## References

[B1] Anders S. Pyl P. T. Huber W. (2015). HTSeq–a Python framework to work with high-throughput sequencing data. Bioinformatics 31, 166–169. 10.1093/bioinformatics/btu638 25260700 PMC4287950

[B2] Berahovich R. D. Miao Z. Wang Y. Premack B. Howard M. C. Schall T. J. (2005). Proteolytic activation of alternative CCR1 ligands in inflammation. J. Immunol. 174, 7341–7351. 10.4049/jimmunol.174.11.7341 15905581

[B3] Berger M. Hsieh C. Y. Bakele M. Marcos V. Rieber N. Kormann M.. (2012). Neutrophils express distinct RNA receptors in a non-canonical way. J. Biol. Chem. 287, 19409–19417. 10.1074/jbc.M112.353557 22532562 PMC3365979

[B4] Calzetti F. Tamassia N. Arruda-Silva F. Gasperini S. Cassatella M. A. (2017). The importance of being “pure” neutrophils. J. Allergy Clin. Immunol. 139, 352.e356–355.e356. 10.1016/j.jaci.2016.06.025 27567327

[B5] Cassatella M. A. (1999). Neutrophil-derived proteins: selling cytokines by the pound. Adv. Immunol. 73, 369–509. 10.1016/S0065-2776(08)60791-9 10399011

[B6] Crepaldi L. Silveri L. Calzetti F. Pinardi C. Cassatella M. A. (2002). Molecular basis of the synergistic production of IL-1 receptor antagonist by human neutrophils stimulated with IL-4 and IL-10. Int. Immunol. 14, 1145–1153. 10.1093/intimm/dxf079 12356680

[B7] Dale D. C. Boxer L. Liles W. C. (2008). The phagocytes: neutrophils and monocytes. Blood 112, 935–945. 10.1182/blood-2007-12-077917 18684880

[B8] Davey M. S. Tamassia N. Rossato M. Bazzoni F. Calzetti F. Bruderek K. (2011). Failure to detect production of IL-10 by activated human neutrophils. Nat. Immunol. 12, 1017–1018. 10.1038/ni.2111 22012430

[B9] Forssmann U. Delgado M. B. Uguccioni M. Loetscher P. Garotta G. Baggiolini M. (1997). CKbeta8, a novel CC chemokine that predominantly acts on monocytes. FEBS Lett. 408, 211–216. 10.1016/S0014-5793(97)00408-0 9187369

[B10] Galani I. E. Andreakos E. (2015). Neutrophils in viral infections: current concepts and caveats. J. Leukoc. Biol. 98, 557–564. 10.1189/jlb.4VMR1114-555R 26160849

[B11] González-Navajas J. M. Lee J. David M. Raz E. (2012). Immunomodulatory functions of type I interferons. Nat. Rev. Immunol. 12, 125–135. 10.1038/nri3133 22222875 PMC3727154

[B12] Griffith J. W. Sokol C. L. Luster A. D. (2014). Chemokines and chemokine receptors: positioning cells for host defense and immunity. Annu. Rev. Immunol. 32, 659–702. 10.1146/annurev-immunol-032713-120145 24655300

[B13] Guan E. Wang J. Roderiquez G. Norcross M. A. (2002). Natural truncation of the chemokine MIP-1 beta /CCL4 affects receptor specificity but not anti-HIV-1 activity. J. Biol. Chem. 277, 32348–32352. 10.1074/jbc.M203077200 12070155

[B14] Hayashi F. Means T. K. Luster A. D. (2003). Toll-like receptors stimulate human neutrophil function. Blood 102, 2660–2669. 10.1182/blood-2003-04-1078 12829592

[B15] Hwang J. Son K. N. Kim C. W. Ko J. Na D. S. Kwon B. S.. (2005). Human CC chemokine CCL23, a ligand for CCR1, induces endothelial cell migration and promotes angiogenesis. Cytokine 30, 254–263. 10.1016/j.cyto.2005.01.018 15927850

[B16] Janke M. Poth J. Wimmenauer V. Giese T. Coch C. Barchet W. (2009). Selective and direct activation of human neutrophils but not eosinophils by Toll-like receptor 8. J. Allergy Clin. Immunol. 123, 1026–1033. 10.1016/j.jaci.2009.02.015 19361845

[B17] Jurk M. Heil F. Vollmer J. Schetter C. Krieg A. M. Wagner H.. (2002). Human TLR7 or TLR8 independently confer responsiveness to the antiviral compound R-848. Nat. Immunol. 3:499. 10.1038/ni0602-499 12032557

[B18] Kasama T. Strieter R. M. Lukacs N. W. Burdick M. D. Kunkel S. L. (1994). Regulation of neutrophil-derived chemokine expression by IL-10. J. Immunol. 152, 3559–3569. 8144935

[B19] Kasama T. Strieter R. M. Standiford T. J. Burdick M. D. Kunkel S. L. (1993). Expression and regulation of human neutrophil-derived macrophage inflammatory protein 1 alpha. J. Exp. Med. 178, 63–72. 10.1084/jem.178.1.63 8315395 PMC2191098

[B20] Love M. I. Huber W. Anders S. (2014). Moderated estimation of fold change and dispersion for RNA-seq data with DESeq2. Genome Biol. 15, 550. 10.1186/s13059-014-0550-8 25516281 PMC4302049

[B21] Matsumoto K. Fukuda S. Hashimoto N. Saito H. (2011). Human eosinophils produce and release a novel chemokine, CCL23, *in vitro*. Int. Arch. Allergy Immunol. 155(Suppl. 1), 34–39. 10.1159/000327263 21646793

[B22] McNab F. Mayer-Barber K. Sher A. Wack A. O'Garra A. (2015). Type I interferons in infectious disease. Nat. Rev. Immunol. 15, 87–103. 10.1038/nri3787 25614319 PMC7162685

[B23] Menten P. Wuyts A. Van Damme J. (2002). Macrophage inflammatory protein-1. Cytokine Growth Factor Rev. 13, 455–481. 10.1016/S1359-6101(02)00045-X 12401480

[B24] Nardelli B. Tiffany H. L. Bong G. W. Yourey P. A. Morahan D. K. Li Y.. (1999). Characterization of the signal transduction pathway activated in human monocytes and dendritic cells by MPIF-1, a specific ligand for CC chemokine receptor 1. J. Immunol. 162, 435–444. 9886417

[B25] Novak H. Müller A. Harrer N. Günther C. Carballido J. M. Woisetschläger M. (2007). CCL23 expression is induced by IL-4 in a STAT6-dependent fashion. J. Immunol. 178, 4335–4341. 10.4049/jimmunol.178.7.4335 17371990

[B26] Patel V. P. Kreider B. L. Li Y. Li H. Leung K. Salcedo T.. (1997). Molecular and functional characterization of two novel human C-C chemokines as inhibitors of two distinct classes of myeloid progenitors. J. Exp. Med. 185, 1163–1172. 10.1084/jem.185.7.1163 9104803 PMC2196270

[B27] Re F. Mengozzi M. Muzio M. Dinarello C. A. Mantovani A. Colotta F. (1993). Expression of interleukin-1 receptor antagonist (IL-1ra) by human circulating polymorphonuclear cells. Eur. J. Immunol. 23, 570–573. 10.1002/eji.1830230242 8436189

[B28] Scapini P. Lapinet-Vera J. A. Gasperini S. Calzetti F. Bazzoni F. Cassatella M. A. (2000). The neutrophil as a cellular source of chemokines. Immunol. Rev. 177, 195–203. 10.1034/j.1600-065X.2000.17706.x 11138776

[B29] Scapini P. Laudanna C. Pinardi C. Allavena P. Mantovani A. Sozzani S. (2001). Neutrophils produce biologically active macrophage inflammatory protein-3alpha (MIP-3alpha)/CCL20 and MIP-3beta/CCL19. Eur. J. Immunol. 31, 1981–1988. 10.1002/1521-4141(200107)31:7<1981::aid-immu1981>3.0.co;2-x 11449350

[B30] Scapini P. Tamassia N. Pucillo C. Cassatella M. A. (2013). Granulocytes and mast cells, in Fundamental Immunology,7th Edn., ed Paul W. E. (Philadelphia, PA: Wolters Kluwer Health; Lippincott Williams & Wilkins), 468–486.

[B31] Smale S. T. Tarakhovsky A. Natoli G. (2014). Chromatin contributions to the regulation of innate immunity. Annu. Rev. Immunol. 32, 489–511. 10.1146/annurev-immunol-031210-101303 24555473

[B32] Son K. N. Hwang J. Kwon B. S. Kim J. (2006). Human CC chemokine CCL23 enhances expression of matrix metalloproteinase-2 and invasion of vascular endothelial cells. Biochem. Biophys. Res. Commun. 340, 498–504. 10.1016/j.bbrc.2005.12.037 16378600

[B33] Tamassia N. Cassatella M. A. (2013). Cytoplasmic receptors recognizing nucleic acids and mediating immune functions in neutrophils. Curr. Opin. Pharmacol. 13, 547–554. 10.1016/j.coph.2013.05.003 23725881

[B34] Tamassia N. Cassatella M. A. Bazzoni F. (2014). Fast and accurate quantitative analysis of cytokine gene expression in human neutrophils. Methods Mol. Biol. 1124, 451–467. 10.1007/978-1-62703-845-4_27 24504968

[B35] Tecchio C. Cassatella M. A. (2016). Neutrophil-derived chemokines on the road to immunity. Semin. Immunol. 28, 119–128. 10.1016/j.smim.2016.04.003 27151246 PMC7129466

[B36] Tracey D. Klareskog L. Sasso E. H. Salfeld J. G. Tak P. P. (2008). Tumor necrosis factor antagonist mechanisms of action: a comprehensive review. Pharmacol. Ther. 117, 244–279. 10.1016/j.pharmthera.2007.10.001 18155297

[B37] Trapnell C. Hendrickson D. G. Sauvageau M. Goff L. Rinn J. L. Pachter L. (2013). Differential analysis of gene regulation at transcript resolution with RNA-seq. Nat. Biotechnol. 31, 46–53. 10.1038/nbt.2450 23222703 PMC3869392

[B38] Viola A. Luster A. D. (2008). Chemokines and their receptors: drug targets in immunity and inflammation. Annu. Rev. Pharmacol. Toxicol. 48, 171–197. 10.1146/annurev.pharmtox.48.121806.154841 17883327

[B39] Votta B. J. White J. R. Dodds R. A. James I. E. Connor J. R. Lee-Rykaczewski E.. (2000). CKbeta-8 [CCL23], a novel CC chemokine, is chemotactic for human osteoclast precursors and is expressed in bone tissues. J. Cell. Physiol. 183, 196–207. 10.1002/(sici)1097-4652(200005)183:2<196::aid-jcp6>3.0.co;2-8 10737895

[B40] Yamashiro S. Kamohara H. Yoshimura T. (1999). MCP-1 is selectively expressed in the late phase by cytokine-stimulated human neutrophils: TNF-alpha plays a role in maximal MCP-1 mRNA expression. J. Leukoc. Biol. 65, 671–679. 10331497 10.1002/jlb.65.5.671

[B41] Yanaba K. Yoshizaki A. Muroi E. Ogawa F. Asano Y. Kadono T.. (2011). Serum CCL23 levels are increased in patients with systemic sclerosis. Arch. Dermatol. Res. 303, 29–34. 10.1007/s00403-010-1078-8 20824279

[B42] Yoshimura T. Takahashi M. (2007). IFN-gamma-mediated survival enables human neutrophils to produce MCP-1/CCL2 in response to activation by TLR ligands. J. Immunol. 179, 1942–1949. 10.4049/jimmunol.179.3.1942 17641061

[B43] Youn B. S. Zhang S. M. Broxmeyer H. E. Cooper S. Antol K. Fraser M. Jr.. (1998). Characterization of CKbeta8 and CKbeta8-1: two alternatively spliced forms of human beta-chemokine, chemoattractants for neutrophils, monocytes, and lymphocytes, and potent agonists at CC chemokine receptor 1. Blood 91, 3118–3126. 9558365

[B44] Zimmermann M. Aguilera F. B. Castellucci M. Rossato M. Costa S. Lunardi C.. (2015). Chromatin remodelling and autocrine TNFα are required for optimal interleukin-6 expression in activated human neutrophils. Nat. Commun. 6, 6061. 10.1038/ncomms7061 25616107

[B45] Zimmermann M. Arruda-Silva F. Bianchetto-Aguilera F. Finotti G. Calzetti F. Scapini P.. (2016). IFNα enhances the production of IL-6 by human neutrophils activated via TLR8. Sci. Rep. 6:19674. 10.1038/srep19674 26790609 PMC4726390

